# Neonatal Feeding Intolerance and Its Characteristics: A Descriptive Study

**DOI:** 10.7759/cureus.29291

**Published:** 2022-09-18

**Authors:** Rawan Khalid Albraik, Emad Shatla, Yosra Mohamed Abdulla, Eman Hussien Ahmed

**Affiliations:** 1 Paediatrics, King Hamad University Hospital, Manama, BHR

**Keywords:** descriptive study, characteristics, abdominal distension, vomiting, neonates, feeding intolerance

## Abstract

Background: Feeding intolerance (FI) is common in preterm infants leading to feeding and gastrointestinal disruptions. FI in newborns can be a sign of a variety of problems, ranging from minor, self-limiting illnesses to serious ones. Many causes are found to cause FI such as low intestinal motility, bacterial colonization, hormonal response, and local immunity response. We aim to find out the characteristics of full-term neonates with FI during the neonatal hospitalization period.

Methods: This is a descriptive study that was conducted at King Hamad University Hospital in Bahrain. Our targeted population included neonates with FI. Full-term neonates (gestational age, GA >36 weeks) with lower than 1 week of age were included. Data were investigated and compared with the mode of delivery.

Results: In this study, 46 neonates were included and their characteristics of FI were evaluated. The most common symptoms observed in neonates with FI were vomiting (91.3%) and abdominal distension (8.7%). Of the total cases, 52.2% of neonates were born through Cesarean section (C-section), while 47.8% had a vaginal delivery. All the neonates had normal APGAR (Appearance, Pulse, Grimace, Activity, and Respiration) scores. C-section cases had more vomiting problems than vaginal delivery cases. Abdominal distention was noted more in neonates born via vaginal delivery when compared to C-section.

Conclusion: During hospitalization after birth, refusal to feed with frequent vomiting or abdominal distension is a common characteristic of FI in neonates. Newborns with this condition require constant monitoring and supportive care from competent nurses.

## Introduction

The newborn infant's capacity to breastfeed by mouth is vital and achieving breastfeeding during the initial phase of life in the maternity ward is a critical responsibility for the infant/mother pair. A full-term newborn should be fed breast milk or formula as early as possible after birth, especially within the first hour after birth [1]. Feeding intolerance (FI) is defined as difficulty to digest enteral feedings and is accompanied by an increase in gastric residuals, abdominal distension, and/or reflux. It is common in preterm infants and commonly results in feeding and gastrointestinal disruptions. FI in newborns can be a sign of a variety of problems, ranging from minor, self-limiting illnesses to serious, life-threatening illnesses [2]. The common cause of FI is low intestinal motility because of prematurity [3]. Enzymatic digestion, bacterial colonization, hormonal response, and local immunity are also possible reasons for FI [4]. FI is very common among preterm infants and its clinical symptoms include abdominal distension, vomiting, bilious gastric residuals, occult or gross bloody stools, and are observed in nearly 29% of such neonates [5]. FI can also occur in full-term babies with those symptoms. For term and preterm newborns, the preferred feeding option is the mother's milk. Its distinct composition of digestive enzymes, probiotics, growth factors, and hormones aids immature gastrointestinal functions while also protecting against microbial changes in the gut [6-7]. Mother's milk feeding is related to better feeding tolerance and may be correlated to a reduction in severe morbidity [6]. The mode of delivery (vaginal or cesarean section) and feeding type (breastfeeding or formula feeding) of neonates are considered the most influential factors in the development of gut microbiota [8]. Even if they are successfully weaned from parenteral nutrition, infants with bowel injuries may still be prone to feeding difficulty due to anatomic changes, dysmotility, and developmental delay, which can affect their ability to feed orally [9]. When treating and evaluating FI, the infant's symptoms and medical history should be considered [10]. Neonates with necrotizing enterocolitis can also exhibit similar symptoms of FI with vomiting, increased and bile-tinged green gastric aspirates, or decreased bowel sounds with abdominal distention and tenderness [9]. This study is aimed at exploring the characteristics of term newborns with FI.

## Materials and methods

Patients and settings

A descriptive study was conducted on neonates at the King Hamad University Hospital. A total of 46 neonates (gestational age, GA >36 weeks) were included. The nurse practitioner's expression of the infant's unwillingness to feed, GA (>36 weeks) with less than 1 week of age, and the presence of FI symptoms was also included. Preterm and term newborns with gastrointestinal anatomic malformations, cleft lip or palate, gastroesophageal reflux disease, sepsis, and respiratory complaints were excluded from the study. The primary goal of this study investigation was to explore FI in neonates, as well as their gravidity parity score and delivery method. The study was conducted following the approval of the Institutional Review Board of King Hamad University Hospital (IRB: 22-530) and consent was taken from the guardian of the neonate.

Study tool and data collection

Data collection covers the following parameters: demographic characteristics, APGAR (Appearance, Pulse, Grimace, Activity, and Respiration) score, gravidity and parity, and mode of delivery. We excluded subjects with missing information on FI symptoms. We further excluded preterm and term newborns with gastrointestinal anatomic malformations, cleft lip or palate, gastroesophageal reflux disease, sepsis, and respiratory complaints from the main analysis. After all these exclusions, the final number of newborns who remained in the study was 46 subjects. Following data collection, all results were then briefly reviewed and coded to ensure the comprehensibility of the results and that the statistical analysis step was appropriately interpreted.

Statistical analysis

All the statistical analysis steps were undertaken using the SPSS Version 26 (IBM Corp., Armonk, NY) statistical package. Means and standard deviations were used to represent the qualitative data, while numbers and percentages were used to represent the categorical data. Parity, gravidity, and FI symptoms were compared with the mode of delivery using cross-tabulation.

## Results

Characteristics of new-born with feeding intolerance

The study was conducted on 46 neonates admitted to King Hamad University Hospital between January 2021 and January 2022. GA ranged from 36 to 42 weeks, with seven neonates at 36-38 weeks. Most of the neonates were in the 38-40 GA group, and the remaining 15 neonates were at 40-42 weeks of GA. The mean birth weight of the included cases was 3.16 ± 0.465. Only one neonate was reported to have an APGAR score of 6, and all the other neonates had a normal 1-min APGAR score. All the neonates had normal APGAR scores (7-10) at 5-min APGAR scores. Of the total cases, 52.2% of neonates were born through C-sections, while 47.8% had a vaginal delivery. For the data description of the current study, parity or gravidity was categorized into four groups (0-1, 2, 3, and 4). In the present study, 28.3% had a gravidity score of 3 and 4 while 32.6% had a parity score of 3, all the studied cases had signs of FI (Table 1). 

**Table 1:** Characteristics of newborns with FI. SD: standard deviation, N: numbers; APGAR: Appearance, Pulse, Grimace, Activity, and Respiration; FI: feeding intolerance

	Variable	N (%)	
Gender	Female		20 (43.5%)
	Male		26 (56.5%)
Gestational age	36-38		7 (15.2%)
	38-40		24 (52.2%)
	40-42		15 (32.6%)
Birth weight (kg)	Mean ± SD		3.16 ± 0.465
APGAR score 1 min	6		1 (2.2%)
	7		2 (4.3%)
	8		22 (47.8%)
	9		21 (45.7%)
APGAR score 5 min	8		2 (4.3%)
	9		22 (47.8%)
	10		22 (47.8%)
Mode of delivery	C-section		24 (52.2%)
	Vaginal delivery		22 (47.8%)
Gravidity	0-1		11 (23.9%)
	2		9 (19.6%)
	3		13 (28.3%)
	4		13 (28.3%)
Parity	0-1		13 (28.3%)
	2		9 (19.6%)
	3		15 (32.6%)
	4		9 (19.6%)

Symptoms of feeding intolerance

**Figure 1:**
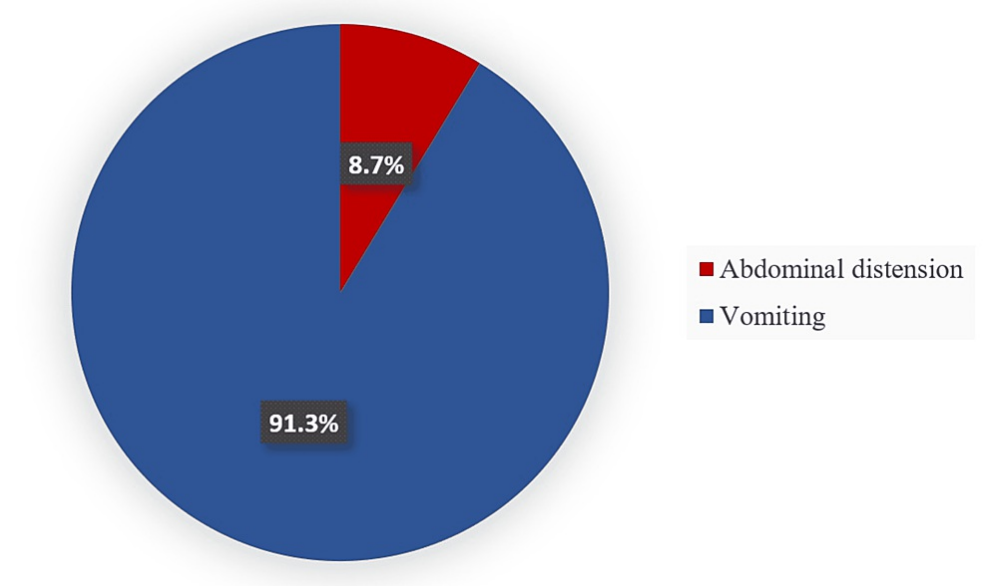
Symptoms of FI. FI, feeding intolerance

The most common symptoms observed in neonates with FI were abdominal distension (8.7%) and vomiting (91.3%). Forty-two of the cases had vomiting and only four had abdominal distension (Figure 1). 

Comparison based on the mode of delivery

When comparing vomiting, C-section cases had more vomiting problems than vaginal delivery cases. Among the four abdominal distension cases, three were vaginal deliveries, and only one had a C-section. It has been observed that the highest gravidity score was observed in mothers who had a C-section and 40.9% of the vaginal delivery cases had a gravidity score of three (Table 2).

**Table 2:** Comparison of gravidity, parity, and FI based on the mode of delivery. FI, feeding intolerance

Variable		C-section	Vaginal delivery
Gravidity	0-1	3 (12.5%)	8 (36.4%)
	2	6 (25%)	3 (13.6%)
	3	4 (16.7%)	9 (40.9%)
	4	11 (45.8%)	2 (9.1%)
Parity	0-1	4 (16.7%)	9 (40.9%)
	2	6 (25%)	3 (13.6%)
	3	6 (25%)	9 (40.9%)
	4	8 (33.3%)	1 (4.5%)
Feeding intolerance	Abdominal distension	1 (0.04%)	3 (0.14%)
	Vomiting	23 (0.96%)	19 (0.86%)

## Discussion

The newborn must quickly adapt from receiving all nutrients through the placenta to receiving them orally after delivery, hence gastrointestinal adaptation is critical. It is a vital stage for the neonate because many organ systems undergo substantial adaptations to meet the extrauterine environment [11-12]. Vomiting in the days following birth is common. In our study, the observed symptoms of FI were recurrent vomiting and abdominal distension. Vomiting was found to be more common than abdominal distension. Moreover, vomiting is considered the main accompanying symptom in early newborns with abdominal distention [13]. Vomiting related to exchange transfusions rarely persists for more than a few hours. The rapidity with which vomiting ceased in babies with gastric irritation was dependent upon the effectiveness with which irritant material was removed. During the early newborn period, however, some infants suffer recurrent vomiting and feeding refusal. Recurrent copious vomiting with reluctance to feed orally is concerning, despite a satisfactory clinical state. We analyzed the characteristics of newborns with FI and compared the characteristics with the delivery method. Vomiting, if interpreted to include regurgitation, is the most common sign encountered in the newly born. Vomiting in the newborn may be due to a specific cause or it may be evidence of general systemic disease. There is general agreement that regurgitation and vomiting in the newborn may be due to errors or difficulties in feeding management, fluid and other material swallowed during delivery, infection, or an intracranial lesion [14]. In 2011, Li et al. explained that gastroesophageal reflux, excessive feeding volume, sepsis, increased intracranial pressure, pyelonephritis, anatomic insults, endocrine derangements inguinal hernia, and inborn errors of metabolism are all among the differential diagnosis of FI with frequent vomiting [14]. With a thorough history and physical examination, it is possible to narrow down a diagnostic path and avoid unneeded testing.

In our study, most of the abdominal distension cases were born through vaginal delivery. However, not many differences were observed. Abdominal distention is a common indicator of FI in newborns and can be life-threatening in severe cases. In addition to congenital megacolon, sepsis was the main cause of abdominal distension in full-term newborns [13]. A study conducted by Boo et al. confirmed that modes of delivery, APGAR score at 1 min, sex, ethnicity, history of resuscitation at birth, birth weight, gestation, and multiple pregnancies were not significantly associated with feed intolerance [15]. This finding correlates with our results. In our study, all the newborns had normal APGAR scores.

Although we described a disease rather than defining risk factors, the study's main weakness was the limited sample size. We believe that the number of newborns in this study is insufficient to conduct statistical testing. To elucidate the factors linked with feeding resistance in neonates, more research in major birth centers and sufficient follow-up data are needed.

## Conclusions

What we found in this study was that during hospitalization after birth in the maternity unit, refusal to feed is a common manifestation of FI. Even though our data was limited, vomiting was the most common manifestation of FI. Furthermore, there were only a few cases of abdominal distension. Further studies are needed on a larger population of neonatal centers to explore those manifestations as they can have very serious complications on neonates.

## References

[1] Gartner LM, Morton J, Lawrence RA, Naylor AJ, O'Hare D, Schanler RJ, Eidelman AI (2005). Breastfeeding and the use of human milk. Pediatrics.

[2] Di Lorenzo C (2020). Approach to the infant or child with nausea and vomiting. Up to Date.

[3] Neu J (2007). Gastrointestinal development and meeting the nutritional needs of premature infants. Am J Clin Nutr.

[4] Cresi F, Maggiora E (2018). Feeding Intolerance and Gastroesophageal Reflux. https://www.researchgate.net/publication/329519563_Feeding_intolerance_and_gastroesophageal_reflux.

[5] Moore TA, Wilson ME, Schmid KK, Anderson-Berry A, French JA, Berger AM (2013). Relations between feeding intolerance and stress biomarkers in preterm infants. J Pediatr Gastroenterol Nutr.

[6] Ford SL, Lohmann P, Preidis GA (2019). Improved feeding tolerance and growth are linked to increased gut microbial community diversity in very-low-birth-weight infants fed mother's own milk compared with donor breast milk. Am J Clin Nutr.

[7] Duale A, Singh P, Al Khodor S (2022). Breast milk: a meal worth having. Front Nutr.

[8] Akagawa S, Tsuji S, Onuma C (2019). Effect of delivery mode and nutrition on gut microbiota in neonates. Ann Nutr Metab.

[9] Neu J, Walker WA (2011). Necrotizing enterocolitis. N Engl J Med.

[10] Duran B (2005). The effects of long-term total parenteral nutrition on gut mucosal immunity in children with short bowel syndrome: a systematic review. BMC Nurs.

[11] Olver RE, Walters DV, M Wilson S (2004). Developmental regulation of lung liquid transport. Annu Rev Physiol.

[12] Burton GJ, Fowden AL (2015). The placenta: a multifaceted, transient organ. Philos Trans R Soc Lond B Biol Sci.

[13] Chen A, Du J, Du LZ (2013). [Clinical characteristics of abdominal distention in early newborns]. Zhongguo dang dai er ke za zhi = Chinese journal of contemporary pediatrics.

[14] Li BU, Sunku BK (2006). Vomiting and Nausea in Pediatric Gastrointenstinal and Liver Disease. Pathophysiology/Diagnosis/Management. 3rd ed.

[15] Boo NY, Soon CC, Lye MS (2000). Risk factors associated with feed intolerance in very low birthweight infants following initiation of enteral feeds during the first 72 hours of life. J Trop Pediatr.

